# Hydrogel–metal-organic-framework hybrids mediated efficient oral delivery of siRNA for the treatment of ulcerative colitis

**DOI:** 10.1186/s12951-022-01603-6

**Published:** 2022-09-05

**Authors:** Meng Gao, Chen Yang, Chenghu Wu, Yue Chen, Hongqin Zhuang, Jilong Wang, Zhiting Cao

**Affiliations:** 1 Department of Gastroenterology, The Second People’s Hospital of Hefei, Hefei Hospital Affiliated to Anhui Medical University, Hefei, 230011 China; 2 School of Biopharmacy, China Pharmaceutical University, Nanjing, 210009 China; 3 Zhejiang Engineering Research Center for Tissue Repair Materials, Wenzhou Institute, University of Chinese Academy of Sciences, Wenzhou, 325000 Zhejiang China; 4 School of Clinical Medicine, Anhui Medical University, Hefei, 230000 China; 5 School of Life Sciences, Nanjing University, Nanjing, 210023 China

**Keywords:** Hydrogel–metal-organic framework hybrids, Delivery system, Sodium alginate, Gastric-intestinal environment, Ulcerative colitis

## Abstract

**Background:**

Ulcerative colitis (UC) is a major type of inflammatory bowel disease (IBD), which could induce bloody stool, diarrhea, colon atrophy and eventually lead to colorectal cancer. The conventional daily oral administration of drugs only relieve the inflammatory response of colon in the short term, Biological agents such as antibody drugs has proven its efficiency in inhibiting colitis, while the low drug bioavailability means that large doses of antibodies are required, ultimately causing systemic toxicity. Small interfering RNA (siRNA) has significant advantages over antibody drugs in terms of safety and efficacy, and it have been widely applied as potential candidates for a variety of inflammation-related diseases. However, oral delivery of siRNA fails to overcome the degradation of the gastrointestinal environment to produce a significant therapeutic effect in ulcerative colitis. Herein, we design the hybrid delivery system that the siRNA loaded MOF encapsulated in the sodium alginate particles to overcome the barriers in the oral process.

**Results:**

The hybrid delivery system (SA@MOF-siRNA^TNFα^) was successfully constructed, and it could not only survive the low pH environment in the stomach and small intestine, but also taken up more by inflammatory macrophages, as well as released much more MOF-siRNA^TNFα^. Moreover, SA@MOF-siRNA^TNFα^ tended to enriched and infiltrated into local colon tissues. As a result, SA@MOF-siRNA^TNFα^ significantly reduced the progression of colitis, of which the treated mice did not experience significant weight loss, bloody stools and diarrhea.

**Conclusion:**

We confirmed that the formulation of hydrogel–metal-organic framework hybrids could improve the protection of incorporated payload in the gastric and early small intestine, enhancing the delivery of MOF-siRNA to colon.

## Background

Ulcerative colitis (UC) is a major type of inflammatory bowel disease (IBD), characterized by inflammatory disorders of colon [1, 2]. Typical symptoms of colitis include weight loss, bloody stool, diarrhea, and colon atrophy. Deformity of physiological function and microenvironment in colon may eventually lead to the development of colorectal cancer [3]. Due to the lack of in-depth understanding of ulcerative colitis, there are no effective methods for the complete cure of colitis. At present, the conventional treatment before surgery is daily oral administration of drugs, such as corticosteroids, aminosalicylic acid, antibiotics, etc. [4]. However, they only relieve the inflammatory response of colon in the short term. Long-term high dose drug usage not only causes drug tolerance, reducing the therapeutic effect, but also the caused serious side effects are not conducive to the health of patients [5]. Biological agents such as antibody drugs prove that it is effective in inhibiting colitis. They has been widely used in the treatment of ulcerative colitis patients [6–8]. However, these antibodies also have their own usage problems, and the low drug bioavailability means that large doses of antibodies are required, ultimately causing systemic toxicity. It is important to find some new treatments to optimize the efficacy for ulcerative colitis while limiting systemic toxicity.

Small interfering RNA (siRNA) has significant advantages over antibody drugs in terms of safety and efficacy [9]. Since the FDA approved the first siRNA drug for the treatment of hereditary transthyretin-mediated amyloidosis in 2018 [10], siRNA drugs have been widely applied as potential candidates for a variety of inflammation-related diseases [11, 12]. There are also numerous preclinical and clinical trials of siRNA therapy for ulcerative colitis [13, 14]. TNFα, as an important pro-inflammatory factor in ulcerative colitis, plays an important role in the occurrence and development of colon inflammation [15]. It has been documented that the patients of serious colitis could be alleviated by the treatment of anti-TNFα siRNA [16–18]. Compared with other treatments for ulcerative colitis, such as intravenous injection and topical administration, oral administration allows patients to deliver drugs to the topical inflammatory tissue by themselves, which has advantages of simple operation and high feasibility. However, as a convenient and important method of administration, the oral utilization of siRNA is very low. Ample evidences demonstrate that the acidic and multi-enzymatic environment of the gastrointestinal tract is detrimental to the stability of siRNA, resulting in its destructive degradation [19, 20]. The current strategy is to use vectors to protect siRNA from contact with the gastrointestinal environment [20, 21]. Murthy et al. fabricated oral thioketal nanoparticles, which delivered siRNA to the localized sites of intestinal inflammation [22]. Du et al. design surface charge tuned PEG-PCL micelle with synthesized lipid for the oral delivery of anti-TNFα siRNA in the colitis mice. However, high loading of siRNA and penetration into the mucosal layer are not fully considered in the current delivery system. Some polymer systems cannot load enough siRNA for their material properties, and the therapeutic effect is mediocre. Partial delivery system successfully release siRNA in the colon, but the siRNA generally cannot penetrate into the thick mucosal layer of inflamed colon and ultimately eliminated into the feces. Therefore, it is very important to develop a delivery system with high siRNA load and high permeability for oral treatment of ulcerative colitis. This is the premise for the clinical transformation of siRNA drugs in ulcerative colitis.

Metal–organic framework (MOF) are formed by coordination between metal ions and organic compounds [23, 24]. Several studies have pointed out that MOF exhibit strong adsorption effect on siRNA [25–27]. They are suitable for the delivery of high siRNA dosage to treat ulcerative colitis. However, MOF is sensitive to the acid and likely to be destroyed when it passes through gastric juice, releasing free siRNA. Sodium alginate has shown the stability and safety of pharmaceutical preparation excipients as a natural polysaccharide [28]. It can protect drug components from damage by the gastrointestinal environment. However, free siRNA released in the colon from sodium alginate particles fail to penetrate the mucosal layer of the inflammatory colon effectively and will be cleared eventually, reducing the therapeutic effect of siRNA. MOF has the potential to permeate the mucosal layer of inflammatory sites, and the only thing that needs to be addressed is degradation in the gastric acid environment [29]. Thus, they were able to complement each other and successfully deliver a high dose of siRNA to the inflammatory colon. Furthermore, the composite system of sodium alginate and MOF has not been reported currently.

**Fig. 1:**
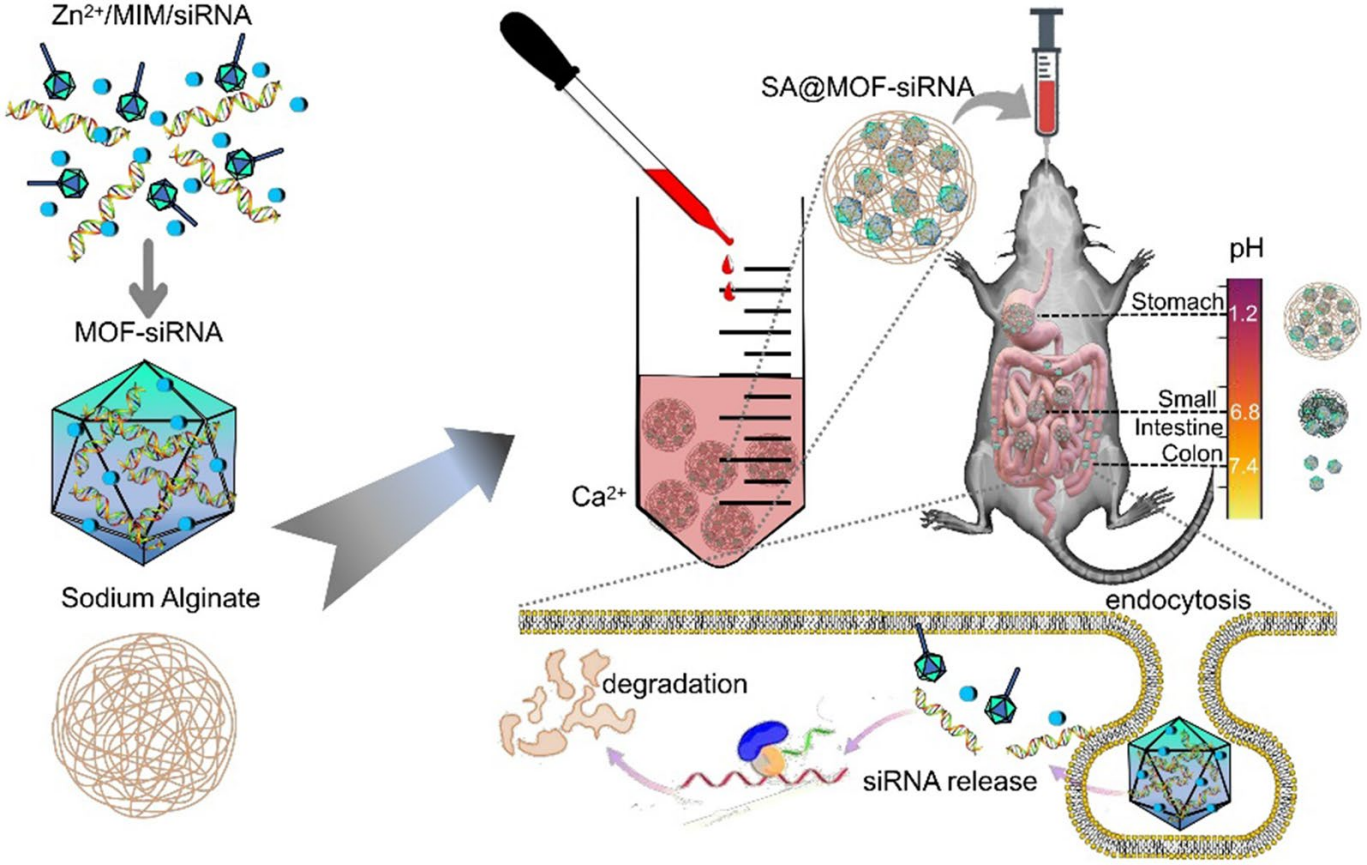
Schematic illustration of the proposed hydrogel–metal-organic framework hybrid delivery system (SA@MOF-siRNA) for the treatment of ulcerative colitis

In this study, we proposed and optimized the use of hydrogel–metal-organic framework hybrid delivery system. siRNA loaded MOF nanoparticles encapsulated in the sodium alginate facilitating the integrity and efficient delivery of the incorporated MOF-siRNA at the site of inflammatory colon. This hybrid delivery system is good at preventing premature drug release from the MOF-siRNA (Fig. 1). Sodium alginate particles could effectively resist the destruction of the payload by gastric and intestinal fluids. Meanwhile, the encapsulated cores were perfectly sized to penetrate the mucosal layer of the colon. The size and morphology of hydrogel–metal-organic framework hybrid delivery system was characterized and the release profiles were assessed in different pH simulated fluid. Furthermore, we examined the accumulation and penetration of this hybrid system in colon and its therapeutic effect on colon inflammation in dextran sulfate sodium (DSS)-induced mouse colitis model.

## Material and methods

### Materials

Fetal bovine serum (FBS) was purchased by ExCell Bio (Guangzhou, China). siTNFα was obtained from Sangon Biotech Co., Ltd. (Shanghai, China). Dulbecco’s minimum essential medium (DMEM) and penicillin streptomycinin were purchased from Gibco (Guangzhou, China). Cell Counting Kit-8 (CCK8) was purchased from Yeasen Biotechnology (Shanghai) Co., Ltd. Sodium alginate (Alg) was purchased from Sigma-Aldrich. Trypsin was purchased from Beijing T&L Biological Technology Co., Ltd. Cell Strainers were obtained from Beijing Solarbio Science and Technology Co., Ltd. Antibodies of DAPI and phalloidineused for immunostaining were purchased from Elabscience Biotechnology Co., Ltd. (Shanghai, China). Zinc nitrate and dimethylimidazole were supplied by SinopharmCo., Ltd. (Hefei, China). Dextran sulphate sodium salt (DSS) was purchased from Beixing biology Co., Ltd. (Hefei, China). Simulated gastric gluid (SGF) and simulated intestinal fluid (SIF) was purchased from LianShuo Biological (Shanghai, China). TNFα elisa kit was purchased from NeoBioscience Technology Co., Ltd. (Shenzhen, China). MPO elisa kit was purchased from Multi sciences Co., Ltd. (Hangzhou, China). Culture plates were purchased from NEST biotechnology (Wuxi, China). All other chemicals in this experiment were purchased from Beijing Solarbio Science and Technology Co., Ltd. All organic solvents were analytical reagent grade and used directly without further purification.

### Fabrication and characterization of SA@MOF

MOF was prepared as the previously reported with some modification of dosage [30]. Briefly, 10 mg of zinc nitrate hexahydrate was dissolved in 1 mL ultrapure water, and 200 mg of 2-methyl imidazole was dissolved in 4 mL ultrapure water. Subsequently, the mixture was stirred for 30 min at 45 ℃. The prepared MOF was purified for 3 times by the centrifugation of 8000 rpm/min. Then, 100 mg of MOF was dispersed into the 5 mL of sodium alginate solution (5%) with the agitation of 30 min. The mixture was added to saturated calcium chloride solution drop by drop through a micro syringe, and homogenized round hydrogel pellets loaded with MOF (SA@MOF) were produced in multiple batches. The prepared hydrogel pellets can be collected by filtering the solution. The siRNA encapsulated hydrogel–metal-organic framework hybrids could be obtained with the addition of siRNA in the fabrication of MOF, crosslinking the sodium alginate into calcium chloride, repeatedly. RhoB (Rhodamine B) labelled MOF were prepared according to the method described above, here, RhoB was covalently bonded to the BSA (bull serum albumin) and then added to the fabrication of MOF. After the collection of MOF-RhoB, the particles were centrifuged to remove the encapsulated RhoB-BSA. The diameters of MOF and SA@MOF were determined by the dynamic light scattering.

### Hydrogel degradation and siRNA release kinetics

For the determination of hydrogel degradation kinetics in the oral delivery, SA@MOF were immersed into the dynamic pH environment from 1.2 to 7.4. At the predetermined time point, the remained SA@MOF were collected for the detection of weight. The degradation profile could be assessed by changes in weight. The evaluation of siRNA release could achieved through the analysis of release medium. The SA@MOF-siRNA were placed into the dialysis bag (Mw = 14,000 kDa) filled with buffer solution of pH from 1.2 to 7.4. Subsequently, the dialysis bag was immersed into the released medium with addition of 0.5% Tween-80. siRNA release was conducted in the shaking water bath at 37 ℃ for 24 h. The released medium was collected with the equal fresh medium instead at the predetermined time. They were centrifuged at 8000 rpm/min for 5 min and then lyophilized to remove the solution. The released siRNA was expressed as the percentage of total siRNA encapsulated in SA@MOF-siRNA.

### RAW264.7 cellular uptake

RAW264.7 macrophage cells were obtained from American Type Culture Collection (ATCC) and cultured at 37 ℃ in the 5% CO_2_ incubator. RAW264.7 cells were seeded in 24-well plates Cells (Jet Bio-Filtration Co., Ltd., Guangzhou, China) at the density of 8 × 10^4^ cells per well for overnight. Then, the cells were treated with different formulations for 8 h. Here, the MOF and SA@MOF were pretreated with simulants (different pH simulated fluid) to simulate oral delivery through the stomach and intestines. The cells were separated by trypsin and collected for flow cytometry analysis.

For the confocal scanning microscope observation (CLSM), the RAW264.7 cells were seeded on the coverslips at the density of 5 × 10^4^ cells per well. The cells were treated with MOF-RhoB and SA@MOF-RhoB. After incubation of 8 h, the supernatant of cells were removed and the cell nuclei were stained by DAPI. Finally, the cells on the coverslips were visualized by the CLSM.

### Cell viability of MOF-siRNA or SA@MOF-siRNA

RAW264.7 cells were seeded into the 96-well plates of 5000 cells per well in 100 μL culture medium. Overnight, the medium were replaced with fresh culture medium containing MOF-siRNA and SA@MOF-siRNA. The amount of total siRNA added to the culture plate was quantified to100 nM. At the predetermined time, the cells were treated with MTT for another 4 h. The cell viability was detected by the microplate readers at the absorbance of 490 nm.

### TNFα quantification in vitro

RAW264.7 cells were maintained in DMEM supplemented with 10% FBS. To evaluate efficacy of knockdown, the RAW264.7 cells activated with lipopolysaccharide (LPS) were seeded in 24-well plates at 1 × 10^5^ cells per well overnight. The cells were then incubated with MOF-siRNA^TNFα^, SA@MOF-siRNA^TNFα^, MOF-siRNA^TNFα^ (pre-treated with simulated fluid) and SA@MOF-siRNA^TNFα^ (pre-treated with simulated fluid) at a siRNA concentration of 100 nM for 24 h. The cells were collected and lysed for the centrifugation at 5000 rpm. The supernatant were detected by the enzyme linked immunosorbent assay (ELISA) for the determination of TNFα in cells.

### In vivo pharmacokinetics and colon accumulation

Female C57BL/6 mice of 6 weeks were obtained from Beijing Vital River Company. All animals were in compliance with the guidelines outlined in the Guide for the Care and Use of Laboratory Animals. The mouse model of colitis were constructed through the administration of 5% DSS orally for 7 days consecutively. The colitis model mice were fasted for 24 h before the experiment. Subsequently, the mice were orally administrated of MOF-RhoB and SA@MOF-RhoB at the dose of 180 μg RhoB. The mice were sacrificed to remove the colon tissue for evaluation of RhoB fluorescence after 12 h. The fluorescence intensity was determined by multimode optical in vivo imaging system (PerkinElmer, America). For the evaluation of pharmacokinetics, the blood was taken from the eye socket of mice. Then, the blood was centrifuged at 300 rpm for 5 min to collect the serum. The pharmacokinetics orally was expressed by the remained RhoB fluorescence in serum. Fluorescence intensity was identified by multimode optical in vivo imaging system.

### Immunofluorescence of colon tissue

The mice were treated as the described above. The colon tissue was removed from the mice after the treatment. Fecal debris in the intestinal tract were washed with PBS buffer for 3 times. Multiple rinses of the colon with 4% paraformaldehyde to fix the shape of the open chamber. Preliminary colonic tissue was then immersed in 4% paraformaldehyde overnight. The colon tissue section of 7 μm was cut by cryosection machine. The cytoskeleton and cell nuclei were stained by Alexa fluor 488 phalloidin and DAPI. They were kept in the anti-fade mounting media to avoid fluorescence photobleaching. Detail information of particles in tissue were assessed by the CLSM.

### Alleviation of ulcerative colitis in vivo

C57BL/6 mice were randomly divided into five groups after a week of adaptation. The mice were treated with different formulations during the 5% DSS administration orally. Among them, one group mice were selected as normal control group without any treatment. Another one was identified as negative control group with only 5% DSS administration. The others received oral treatment of SA@MOF-siNC, MOF-siRNA^TNFα^and SA@MOF-siRNA^TNFα^ with the siRNA dosage of 5 nmol per mouse every day. Meanwhile, the weight of mice were recorded and disease activity index were judged according to the mice behaviors. The mice were sacrificed to remove the colon and spleen for assessment at the last day of treatment. The supernatant of colon tissue was collected by homogenization and centrifugation for quantitative detection of MPO and TNFα. In addition, the changes in systematic inflammation were determined by TNFα in serum from treated mice.

### Statistical analysis

Analyses were performed on GraphPad Prism 8.0. The data of results were shown as mean ± standard deviation (SD). The statistical analysis was performed using Student’s t-test or one-way analyses of variance (ANOVA). Values with P < 0.05 were defined statistically significant (n.s means no significance, *means P < 0.05, ** means P < 0.01, *** means P < 0.001, **** means P < 0.0001).

## Results and discussion

### Preparation and characterization of particles

**Fig. 2:**
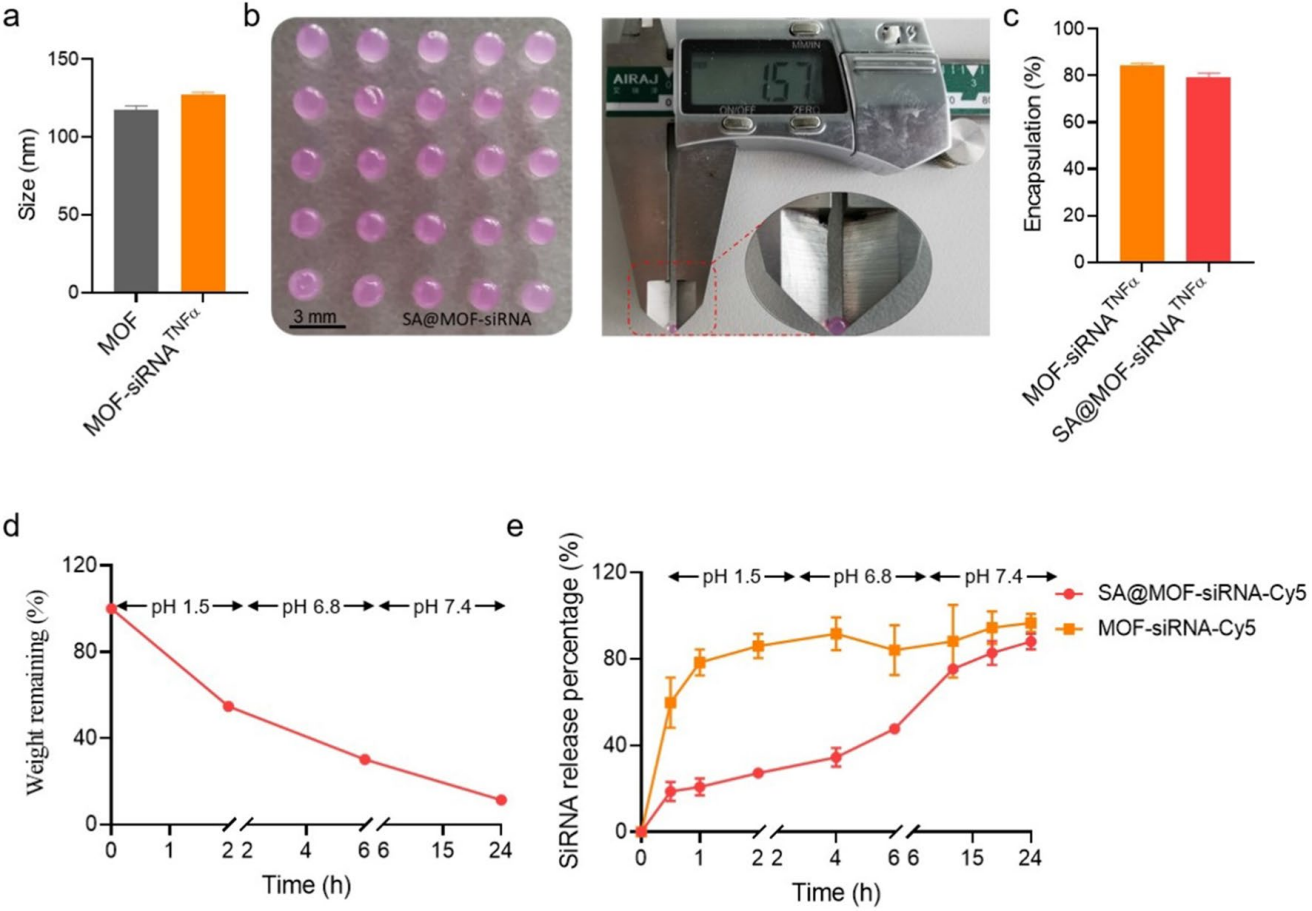
Physicochemical characterization of MOF-siRNA and SA@MOF-siRNA. Diameters of MOF and MOF-siRNA^TNFα^ (**a**). Optical image and size of SA@MOF-siRNA (**b**). siRNA encapsulation of MOF-siRNA and SA@MOF-siRNA (**c**). Degradation of SA@MOF-siRNA in the gastrointestinal tract simulation fluid (pH arranged from 1.5 to 7.4) (**d**). The released profile of siRNA from MOF-siRNA and SA@MOF-siRNA in the SIF/SGF (**e**)

The MOF and MOF-siRNA were prepared as described in the section of Methods. Their average sizes detected by dynamic light scattering were around 117.7 nm and 127.2 nm, respectively (Fig. 2a). The results showed that the size of MOF increased slightly with the incorporation of siRNA. Furthermore, Sodium alginate hydrogel–metal-organic framework hybrids were fabricated with MOF-RhoB instead according to the instruction in Methods. The prepared SA@MOF-RhoB could be visualized and exhibited crystal ball. The diameter detected by vernier calipers was 1.57 mm (Fig. 2b). The red sheen in the spheres also indicated that the MOF particles were successfully encapsulated in the hydrogel. The encapsulation rates of siRNA in MOF-siRNA and SA@MOF-siRNA reached 84.2% and 79.3%, respectively (Fig. 2c). It demonstrated that the formulation of sodium alginate hydrogel–metal-organic framework hybrids could well keep siRNA loading without worrying about the amount of siRNA. The stability of hydrogel in gastroenteric fluid was investigated to confirm the prevention of inside MOF-siRNA. The SA@MOF-siRNA was immersed into the simulation fluid with different pH for certain time according to the gastrointestinal retention time after oral administration [31]. The weight of SA@MOF-siRNA dropped from 100% to 54.71% after 2 h in buffer pH 1.5. Then, the hybrids were treated with buffer pH 6.8 for another 4 h, dropping to 30.2%. Subsequently, the remained SA@MOF-siRNA was transferred to the buffer pH 7.4 for 18 h, leaving 11.3% of particles (Fig. 2d). These results suggested that sodium alginate hydrogel shells could delay the degradation in gastroenteric fluid, but failed to reverse the fate. Another way, it was also conducive to the release of contents. However, whether it affected the stability of siRNA remained to be further investigated. The release profile indicated that siRNA could be released slowly and continuously for a long time with the protection of sodium alginate hydrogel shells. There were still 52.3% intact siRNA in the SA@MOF-siRNA after the destruction of gastric juice and small intestine fluid. By contrast, 84.12% of siRNA had been released from MOF-siRNA (Fig. 2e). This suggested that fewer siRNA could reach the inflammatory site of colon. According to the preliminary experimental design, we hoped to protect the siRNA by sodium alginate hydrogel from being damaged by gastric and intestinal fluid, then more MOF-siRNA particles could be released in the colon and help siRNA to pass through the mucosal layer and delivered to inflammatory macrophages.

### In vitro cellular uptake

**Fig. 3:**
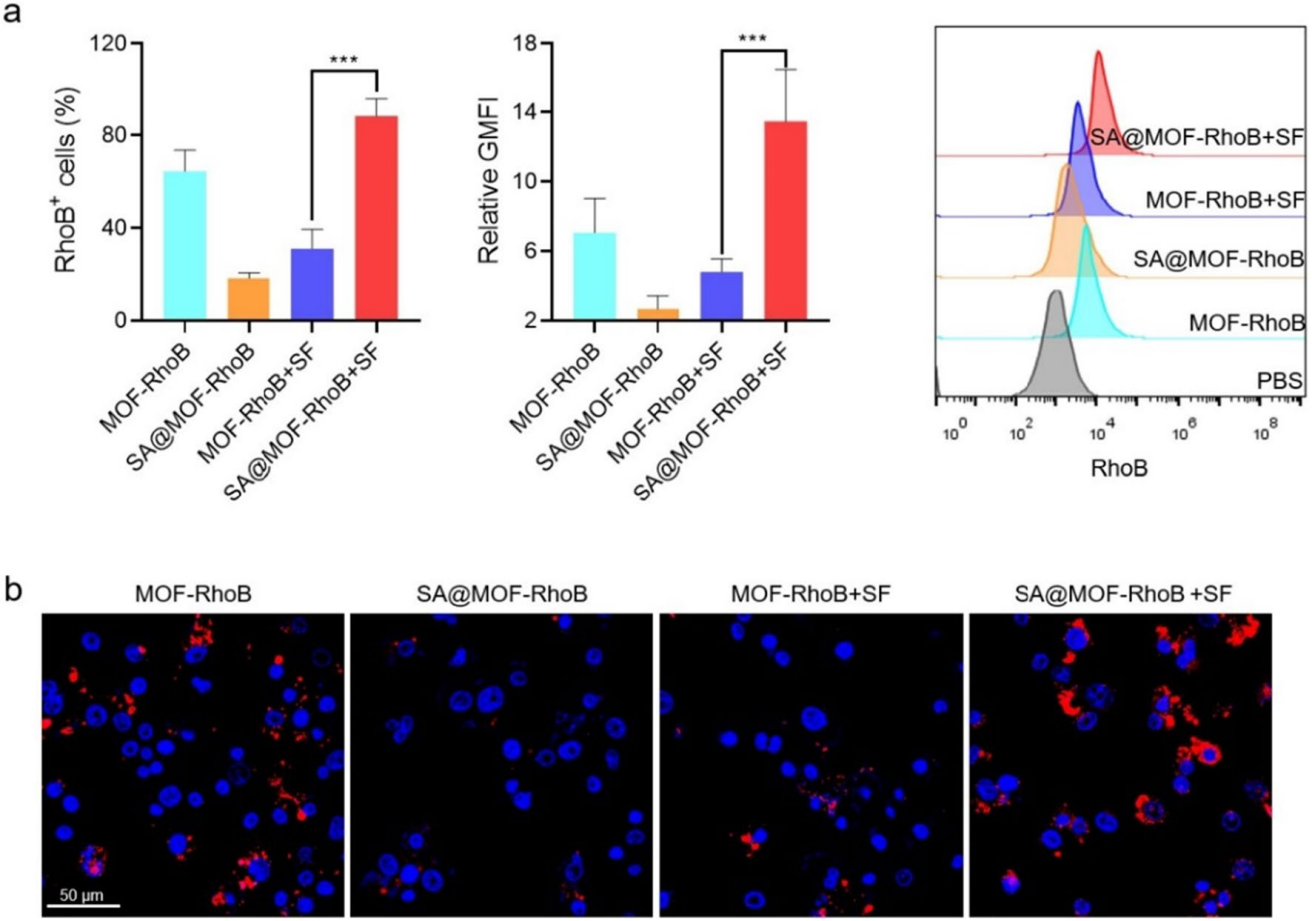
Cellular uptake of MOF-RhoB and SA@MOF-RhoB by RAW264.7 cells. **a** RhoB fluorescence intensity of RhoB-positive RAW264.7 cells detected by flow cytometry. **b** The RhoB fluorescence in RAW264.7 cells with the treatment of different formulations by CLSM. MOF-RhoB + SF indicated that MOF-RhoB treated with gastrointestinal tract simulation fluid (pH value arranged from 1.5 to 7.4). SA@MOF-RhoB + SF indicated that SA@MOF-RhoB treated with gastrointestinal tract simulation fluid (pH arranged from 1.5 to 7.4). The blue indicated the cell nucleus. ***P < 0.001

A large number of inflammatory macrophages infiltrated into ulcerative colon, which aggravated the deterioration of inflammation by secreting inflammatory factors such as TNFα [32, 33]. To assess the difference in cellular uptake, RAW264.7 cells were treated with MOF-RhoB and SA@MOF-RhoB. Meanwhile, RAW264.7 cells of both groups were incubated with the particles, which were pre-treated with gastrointestinal simulation fluid. We believed it was a good way to give feedback on what's going on in the body. The results in Fig. 3a demonstrated that the cellular uptake of SA@MOF-RhoB pre-treated with gastrointestinal simulation fluid was higher than others. This is because under the treatment of gastrointestinal simulation fluid, the thick biomineralized layer on the particles surface were degraded, releasing the inside MOF-RhoB particles and promoting the uptake of fluorescent particles by RAW264.7 cells. Meanwhile, the millimetre size of SA@MOF-RhoB were not conductive to the intake without treatment. The geometric mean fluorescence intensity also exhibited similar results. The fluorescence intensity of RhoB in SA@MOF-RhoB pre-treated with gastrointestinal simulation fluid group was stronger than others (Fig. 3b). Results also showed the advantage of SA@MOF-RhoB for oral delivery of siRNA in vivo.

### In vitro inhibition of siTNFα

**Fig. 4:**
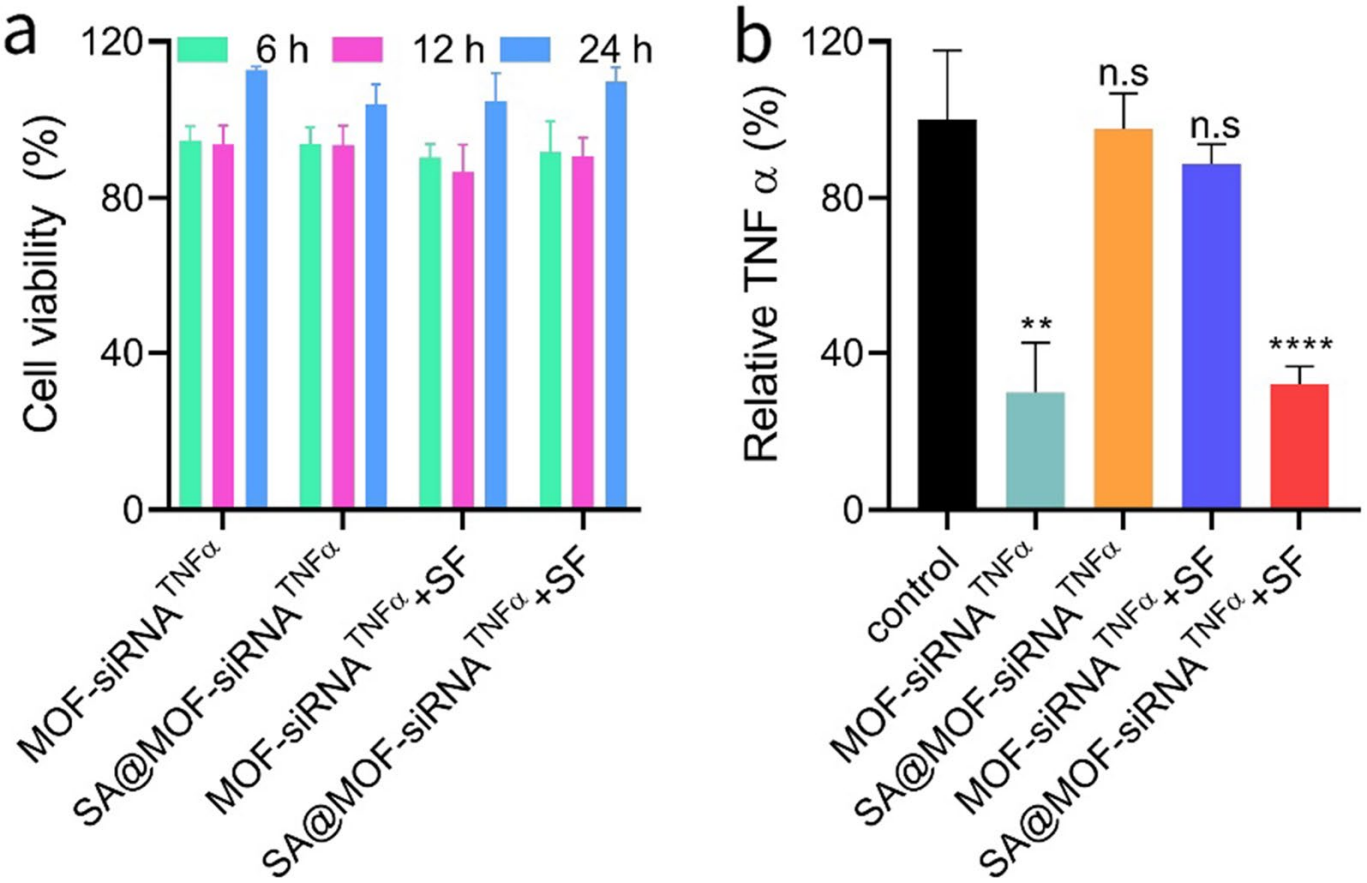
Cell viability and siTNFα inhibition of MOF-siRNA^TNFα^ and SA@MOF-siRNA^TNFα^. **a** Cytotoxicity of RAW264.7 cells treated with different formulations. **b** Quantitative analysis of TNFα in RAW264.7 cells by Elisa kit. n.s means no significance, **P < 0.01, ****P < 0.0001

To ensure safety of particles for cells, RAW264.7 cells were incubated with different siTNFα formulations for 6 h, 12 h and 24 h. The cells were added with cck8 reagent for another 4 h and then detected by the microplate readers. Results in Fig. 4a demonstrated that there was no cytotoxicity for different particles. To further evaluate the inhibitory efficiency of siTNFα, the cells treated with different siTNFα formulations were collected for quantitative analysis. The inhibitory efficiency of SA@MOF-siRNA^TNFα^ pre-treated with different pH gastrointestinal tract simulation fluid was significantly better than that of others’ (Fig. 4b). However, the MOF-siRNA^TNFα^ pre-treated with gastrointestinal tract simulation fluid failed to exhibit good inhibitory efficiency. The acid sensitivity of MOF was not conducive to the protection of siRNA in gastroenteric fluid, resulting in low inhibitory effect. These results also indicated the advantages of sodium alginate hydrogel and MOF hybrid particles in oral drug delivery.

### In vivo colon accumulation and blood circulation

**Fig. 5:**
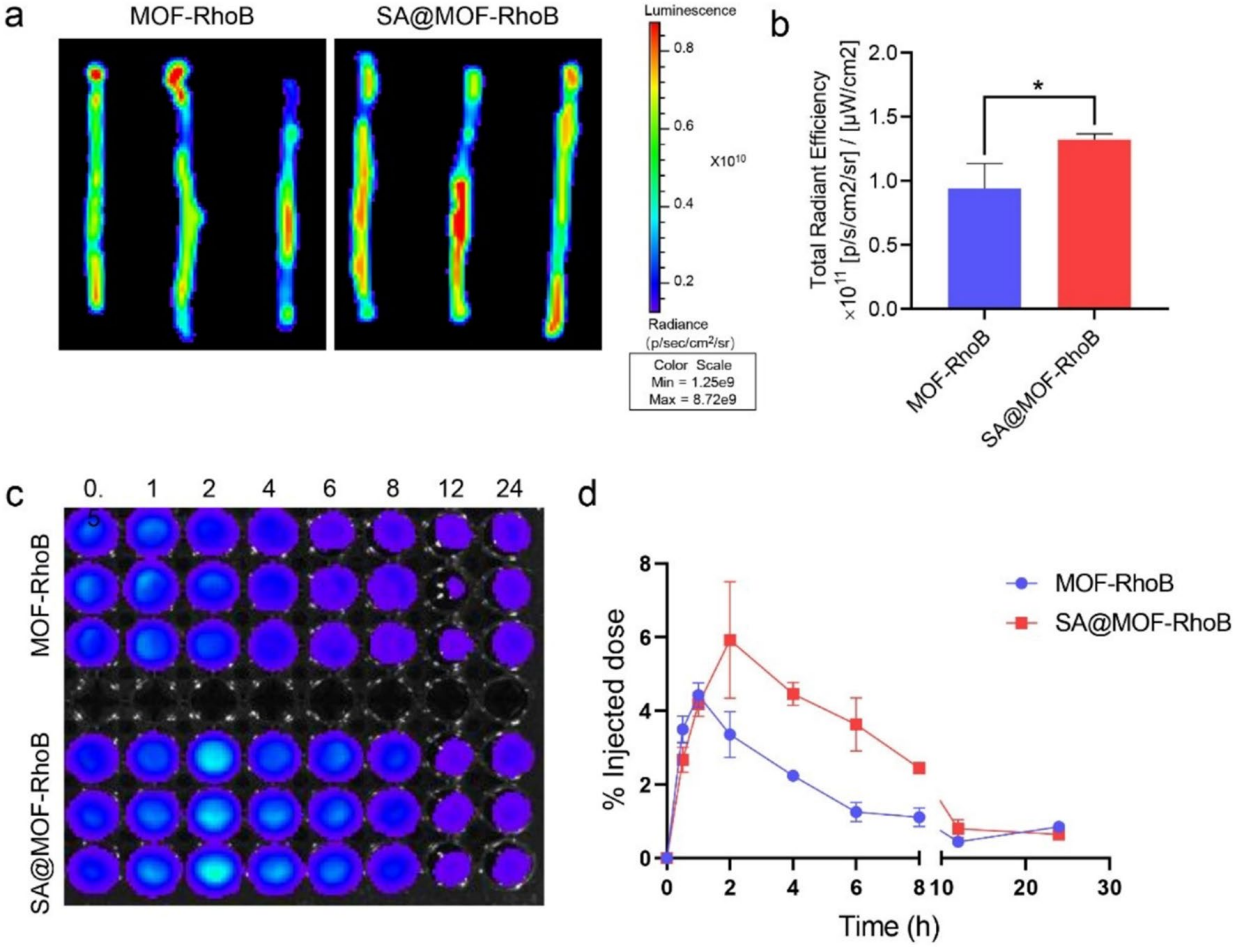
Colon accumulation and blood circulation of MOF-RhoB and SA@MOF-RhoB. **a** Biodistribution of particles in colon detected by multimode optical in vivo imaging system. **b** Statistic fluorescence intensity. **c** RhoB intensity identified in serum. **d** Changes of fluorescence intensity in serum over time. *means P < 0.05

The mice were induced for ulcerative colitis according to the usage of DSS [34, 35]. Then, the diseased mice were orally administrated of RhoB labeled MOF-RhoB and SA@MOF-RhoB. After 12 h, the mice were sacrificed to remove the colon tissue. The feces in colon were washed three times with PBS. Subsequently, the colon was imaged by the multimode optical in vivo imaging system. It could obviously be found that the intensity of fluorescence signal in SA@MOF-RhoB was stronger than MOF-RhoB (Fig. 5a). Statistical results of fluorescence also showed that there were significantly more SA@MOF-RhoB in the colon than MOF-RhoB (Fig. 5b). As far as we know, the intestinal tract has the function of absorbing into the blood, and the particles that enter the blood still have the effect of controlling inflammation. Therefore, we collected the blood from the treated mice at different time points to determine the ability of the two particles to enter the blood through the intestinal tract. The imaging results showed that the amount of SA@MOF-RhoB in the blood was much higher than that of MOF-RhoB (Fig. 5c). It implied that SA@MOF-RhoB has longer circulation time through oral administration. Furthermore, the statistical results more directly supported this conclusion (Fig. 5d).

### Particles penetration in colon

**Fig. 6:**
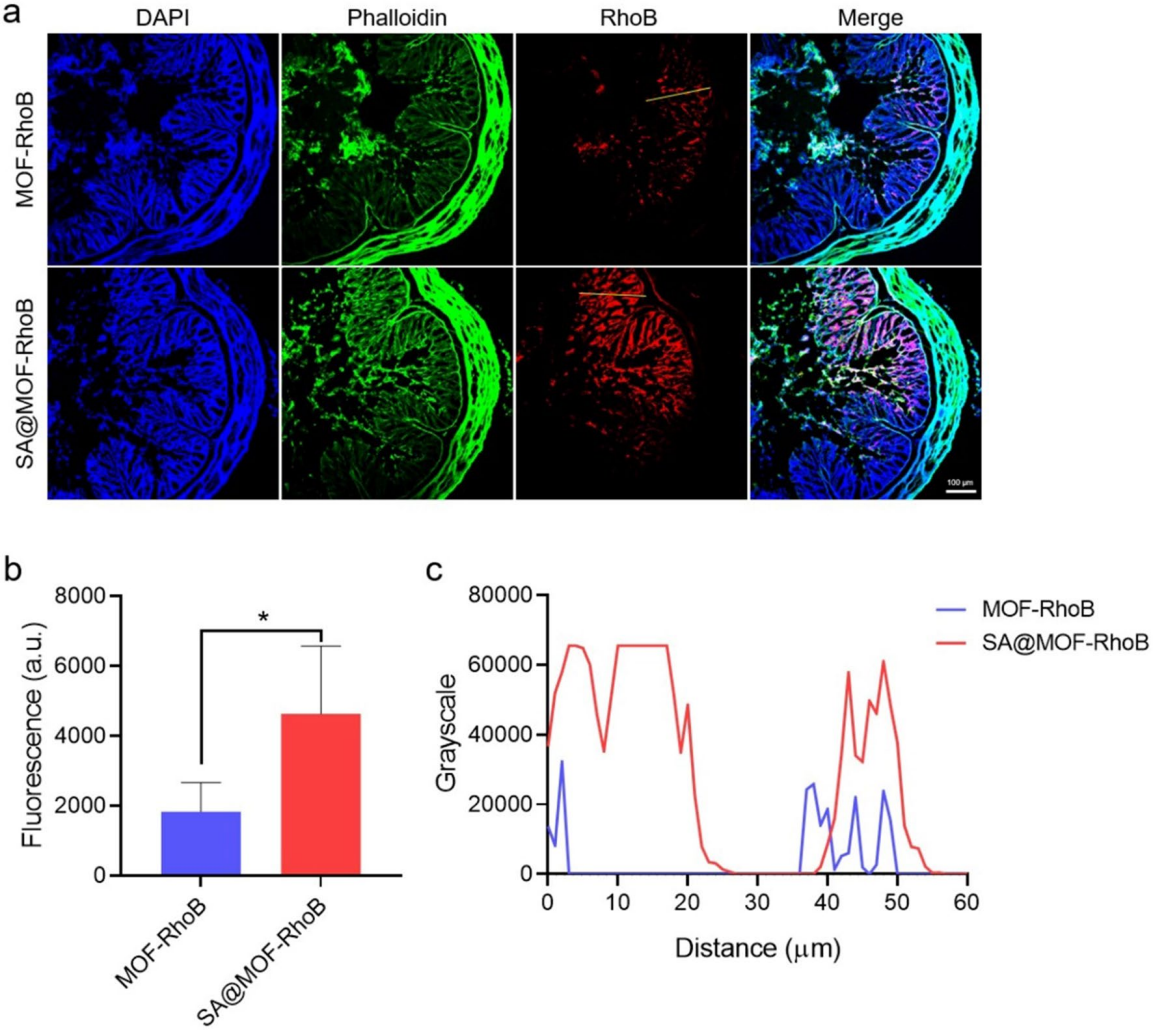
Penetration of MOF-RhoB and SA@MOF-RhoB in colon. **a** Immunofluorescence of colon tissue detected by CLSM. **b** RhoB fluorescence statistics of colon tissue sections. **c** Fluorescence distribution in linear area of colon tissue. The yellow line is the statistical area from the lumen to lamina propria. *means P < 0.05

It has been proved that SA@MOF-RhoB was more abundant in the colon than MOF-RhoB. This was a prerequisite for the control of local colon inflammation, and penetration was the key point for good therapeutic effect. To evaluate the penetration, the diseased mice were oral administrated with MOF-RhoB and SA@MOF-RhoB. The colon tissues were collected from the mice, and for the preparation of frozen tissue section. The immunofluorescence results exhibited that the RhoB florescence in SA@MOF-RhoB group was stronger than that of MOF-RhoB group. In addition, fluorescence was more deeply distributed from the colon lumen to lamina propria (Fig. 6a). Local fluorescence statistics of colon section also showed that RhoB labeled particles of SA@MOF-RhoB group were more easily enriched in colon tissue (Fig. 6b). Meanwhile, we fitted the fluorescence changes from colonic lumen to lamina propria, which indicated the drug could escape the retention of the colon mucosa and enter the interior. The results in Fig. 6c revealed that the SA@MOF-RhoB still could deliver into the colon interior through gastrointestinal environment. We speculated that the sodium alginate hydrogel was damaged by the gastrointestinal tract, releasing MOF-RhoB, which penetrated the mucosal layer. However, MOF-RhoB were easily destroyed by the acidic stomach environment, and the released RhoB was quickly removed.

### In vivo therapeutic efficacy

**Fig. 7:**
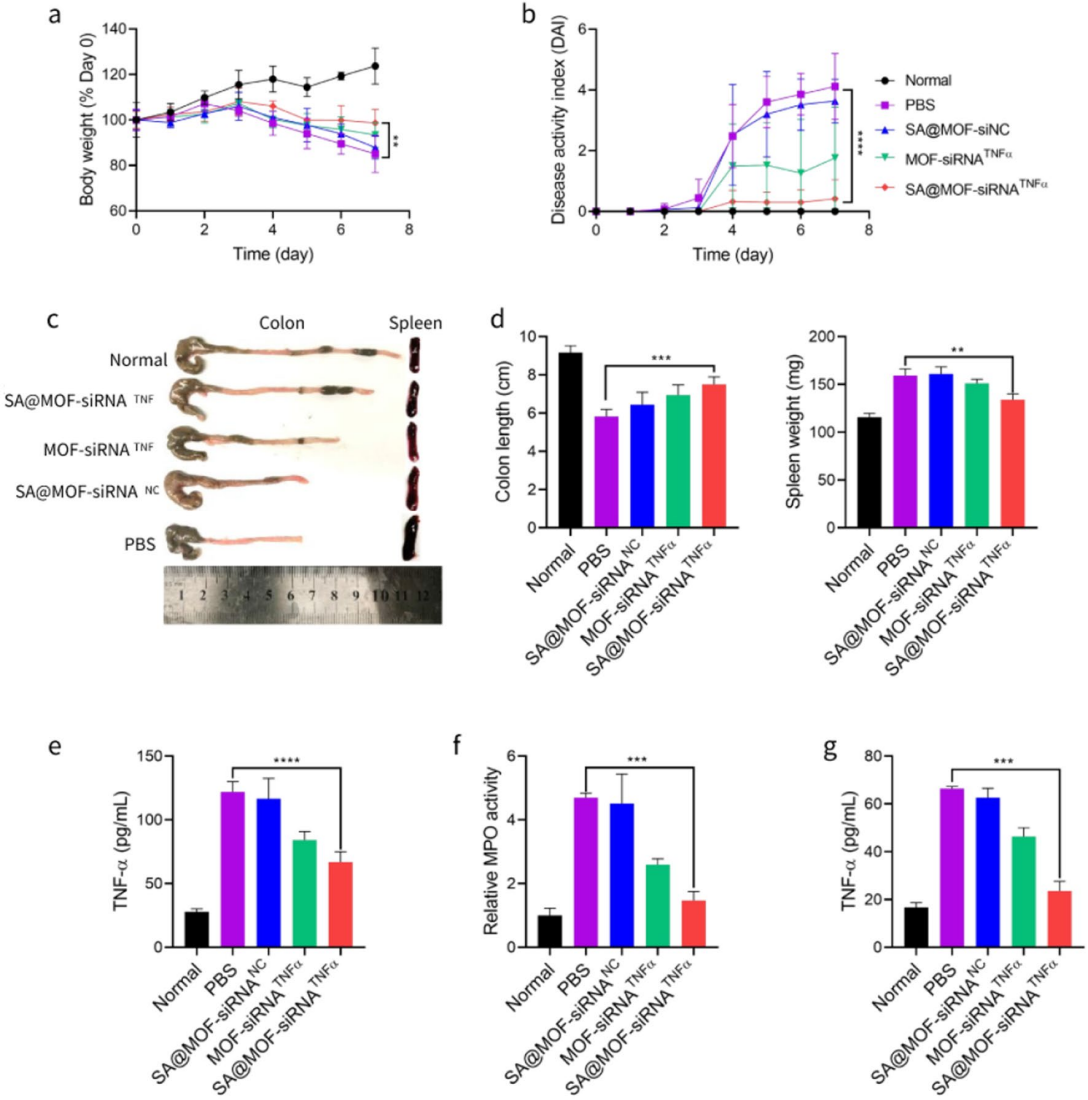
Assessment of ulcerative colitis in mice after oral administration of MOF-siRNA^TNFα^ and SA@MOF-siRNA^TNFα^. **a** Changes in the body weight of mice with the normalized as a percentage of initial body weight. **b** DAI scores. **c** Colon and spleen images. **d** Colon length and spleen weight. **e** Levels of TNFα and **f** myeloperoxidase in mice colon tissue via Elisa. **g** Levels of TNFα in mice serum. **P < 0.01, ***P < 0.001, ****P < 0.0001

Mice models of ulcerative colitis could be easily induced by DSS. This model has been extensively used for the evaluation of treatment in ulcerative colitis [35]. The C57BL/6 mice were randomly assigned to different groups with administration of DSS in drinking water. Some groups of mice received treatment from the next day. During the treatment, we recorded and observed common parameters of ulcerative colitis, such as bloody stool, body weight loss. The results in Fig. 7a showed that the body weight of mice treated with normal water increased steadily. However, DSS treated mice lost 15% of their initial body weight within seven days. The mice treated with SA@MOF-siRNA^TNFα^ remained well within 98.6% of their initial weight. Meanwhile, we also observed the mice biological behavior, and calculated disease activity index (DAI) for evaluating the severity of inflammation. For normal mice, the DAI kept zero every day, indicated there was no obvious inflammatory response. The DSS treated mice with no treatment achieved high scores, while the mice treated with SA@MOF-siRNA^TNFα^ were controlled at a low level (Fig. 7b). Furthermore, the mice were sacrificed to remove the colon and spleen. The colon length was measured by ruler. The colons of DSS treated mice showed varying degrees of atrophy compared with the normal group. The SA@MOF-siRNA^TNFα^ treated mice exhibited the least colon atrophy. The weight of mice spleens showed similar results (Fig. 7c and d). The TNFα in colon tissue also reflected the difference of colon inflammation. The determination of TNFα confirmed the better therapeutic effect (Fig. 7e). Meanwhile, we also measured the myeloperoxidase (MPO) in colon. Fewer inflammatory cytokines were produced in the SA@MOF-siRNA^TNFα^ treated mice (Fig. 7f). The quantitative results of TNFα in serum also indicated that the SA@MOF-siRNA^TNFα^ not only controlled local colon inflammation, but also effectively avoided systemic inflammation (Fig. 7g). These results suggested that oral delivery of gel-MOF hybrid particles was an ideal carrier for colonic inflammation and contributed to systemic control of inflammatory response.

## Conclusion

In the present study, we generated siRNA^TNFα^ loaded sodium alginate hydrogel and metal–organic framework hybrid particles (SA@MOF-siRNA^TNFα^). It could be observed that homogenous MOF-siRNA^TNFα^ distributed into the sodium alginate microspheres. MOF exhibited strong encapsulating effect on siRNA, and its loading rate did not decrease for the encapsulation of hydrogel. The siRNA releasement profile from the SA@MOF-siRNA^TNFα^ was assessed in different pH buffer corresponding to different microenvironment in the gastrointestinal tract. MOF-siRNA^TNFα^ were unable to resist acidic environments and rapidly released large amounts of siRNA. By contrast, SA@MOF-siRNA^TNFα^ could safely survive the low pH environment in the stomach and small intestine because of its gel protection. Furthermore, the released MOF-siRNA^TNFα^ from the SA@MOF-siRNA^TNFα^ could be more taken up by inflammatory macrophages in vitro. The low TNFα expression also demonstrated that SA@MOF-siRNA^TNFα^ system could deliver more siRNA^TNFα^ to macrophages after the destruction of gastrointestinal simulation fluid. In vivo oral delivery experiment, we found that more target drugs were enriched in colon tissues from SA@MOF-siRNA^TNFα^ group via the detection of multimode optical in vivo imaging system. Meanwhile, immunofluorescence results from tissue sections showed that target drugs from SA@MOF-siRNA^TNFα^ group exhibited stronger infiltration effect in local colon tissues. In the alleviation experiment of colitis model mice, SA@MOF-siRNA^TNFα^ could significantly reduce the progression of colitis. The treated mice did not experience significant weight loss, bloody stools and diarrhea. These results indicated the composite microspheres formed by sodium alginate gel and metal–organic framework could effectively protect siRNA drugs from destruction of low pH of gastroenteric fluid, and released MOF-siRNA^TNFα^ nanoparticles in the colon, which were enriched and penetrated into the colon, inhibited the TNFα production of inflammatory macrophages, and ultimately reduced the inflammatory response of colon. We believed that this oral delivery system could effectively solve the defects of RNA drugs in the treatment of ulcerative colitis and improve the therapeutic effect, which broaden the current clinical treatment methods and extended to the treatment of advanced enteritis and even colorectal cancer.

## Data Availability

All relevant figures and tables are included in this manuscript.
